# Stiff Knee Gait Disorders as Neuromechanical Consequences of Spastic Hemiplegia in Chronic Stroke

**DOI:** 10.3390/toxins15030204

**Published:** 2023-03-07

**Authors:** Sheng Li

**Affiliations:** 1 Department of Physical Medicine and Rehabilitation, McGovern Medical School, University of Texas Health Science Center—Houston, Houston, TX 77030, USA; sheng.li@uth.tmc.edu; Tel.: +713-797-7125; Fax: +713-797-5261; 2 TIRR Memorial Hermann Research Center, TIRR Memorial Hermann Hospital, Houston, TX 77030, USA

**Keywords:** spasticity, gait, stroke, botulinum toxin, nerve block

## Abstract

Stiff knee gait (SKG) is defined as decreased knee flexion during the swing phase. It is one of the most common gait disorders following stroke. Knee extensor spasticity is commonly accepted as the primary cause. Clinical management has focused on the reduction in knee extensor spasticity. Recent advances in understanding of post-stroke hemiplegic gait suggest that SKG can present as mechanical consequences between muscle spasticity, weakness, and their interactions with ground reactions during walking. Various underlying mechanisms are presented through sample cases in this article. They include ankle plantar flexor spasticity, knee extensor spasticity, knee flexor and extensor coactivation, and hip flexor spasticity. Careful and thorough clinical assessment is advised to determine the primary cause for each patient. Understanding of these various presentations of SKG is helpful to guide clinical assessment and select appropriate target muscles for interventions.

## 1. Introduction

Stiff knee gait (SKG) is defined as reduced knee flexion during the swing phase [1]. SKG is one of the most common gait disorders following stroke. It affects approximately 60% of stroke patients with gait disorders [2]. In stroke survivors with SKG, it is commonly observed in clinical practice that, to compensate for the reduced knee flexion during the swing phase, ipsilateral hip circumduction or contralateral vaulting occurs to clear the foot [1]. The pattern of walking compensation is also reflected in the knee position in the mid stance phase. The knee joint is either into hyperextension, relying on the hip extensor muscle strength and passive knee stability, or into flexion, shifting the demand to the quadriceps muscle [3]. The adopted compensatory walking pattern increases energy expenditure and decreases the risk of falling as well.

The pathophysiology of SKG is not fully understood. It is mostly accepted that spasticity, or overactivity of the quadriceps femoris muscle, particularly the rectus femoris (RF) is the primary cause of SKG [1,4,5,6,7,8]. RF overactivity is associated with increased knee extension moment in the swing phase and decreased knee flexion velocity at the toe-off. Both potentially contribute to decreased knee flexion. Clinical management of problematic SKG has focused on spasticity reduction in the quadriceps muscles. Interventions include botulinum toxin injections [9,10,11], phenol nerve blocks [7], and surgical quadriceps lengthening [12]. Among these interventions, botulinum toxin injections are well studied, and showed significant improvement in peak knee flexion swing. However, its effect on muscles and functional outcome, such as walking speed and energy expenditure, remains unclear [11].

Recent advances in understanding of post-stroke hemiplegic gait suggest that SKG can present as mechanical consequences between muscle spasticity, weakness and their interactions with ground reactions during walking [13]. SKG can occur as a result of spasticity in other muscles, muscle weakness, and abnormal muscle coactivation of knee flexors and extensors or in any combination of these impairments. Spasticity in ankle plantar flexors can place the ankle into plantar flexion and subsequently force the knee into hyperextension, thus limiting knee flexion during the swing phase [14]. Decreased strength of the hip flexors and ankle plantar flexors lead to decreased power to propel the leg into the swing [15,16,17,18]. Increased knee co-contraction during walking in chronic stroke survivors with SKG might be an adaptive strategy to increase walking stability [19].

Understanding different mechanisms and various clinical presentations of SKG is essential to selecting appropriate management options for the underlying cause. Common causes of SKG are presented here through sample cases. They include ankle plantar flexor spasticity, knee extensor spasticity, knee flexor and extensor coactivation, and hip flexor spasticity.

## 2. Common Mechanisms of Stiff Knee Gait

### 2.1. Ankle Plantar Flexor Spasticity

**Figure 1:**
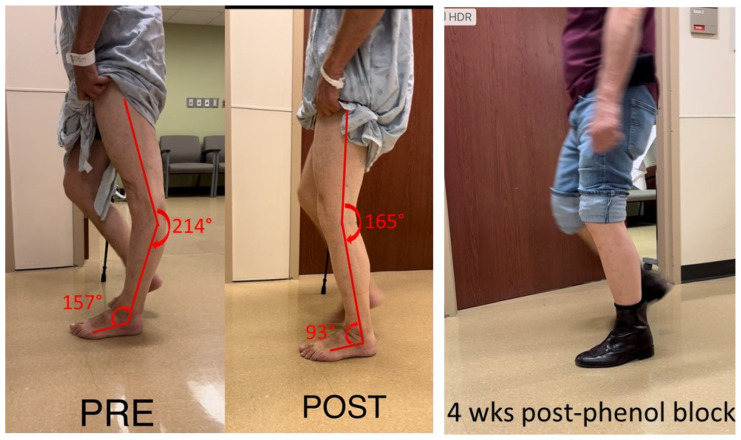
Knee hyperextension due to ankle plantar flexor spasticity (PRE). Joint alignment is corrected after lidocaine block to the tibial nerve (POST). The correction is maintained after phenol nerve block to the tibial nerve (4 wks post-phenol block).

This case examined a 75-year-old male with hemiplegia and abnormal gait as the result of a stroke 25 years ago. He ambulated with a point cane for safety. On the physical examination, manual muscle testing (MMT) grades in the left leg were 3/5 for hip flexors, 4/5 for hip extensors, 5/5 for knee extensors, 3/5 for hamstrings muscles, 4/5 for ankle plantar flexors, and 3/5 for ankle dorsiflexors. Modified Ashworth scale (MAS) assessment showed 2 for ankle plantar flexors, 1 for flexor digitorum longus muscles, 1 for tibialis posterior muscles, and 0 for proximal muscles (hip flexors, extensors, knee flexors and extensors). Left patellar tendon reflex was hyperreflexive, but there was no ankle clonus. During the swing phase, he had mild left hip hiking and circumduction, ankle planar flexion, and minimum knee flexion. During the stance phase, as shown in Figure 1, he had hip extension, knee hyperextension and ankle plantarflexion. Excessive ankle plantarflexion resulted from ankle plantar flexor spasticity shifted the center of gravity anterior to the knee joint. Hip flexor weakness made it difficult to flex the hip joint. After a 1% lidocaine diagnostic injection to the tibial nerve, his ankle plantar flexor spasticity was diminished, and the ankle plantarflexion abnormality was corrected. Subsequently, his left knee hyperextension was corrected during the stance phase (Figure 1 POST). However, he still had the same difficulty in flexing hip and knee during the swing phase due to muscle weakness. On a different day, his tibial nerve motor branches to medial and lateral gastrocnemius and soleus muscles were blocked with 6% phenol. During a 4-week follow up visit, his left hip, knee, and ankle alignment were maintained. He was able to ambulate without a cane, i.e., functional improvement.

### 2.2. Knee Extensor Spasticity

**Figure 2:**
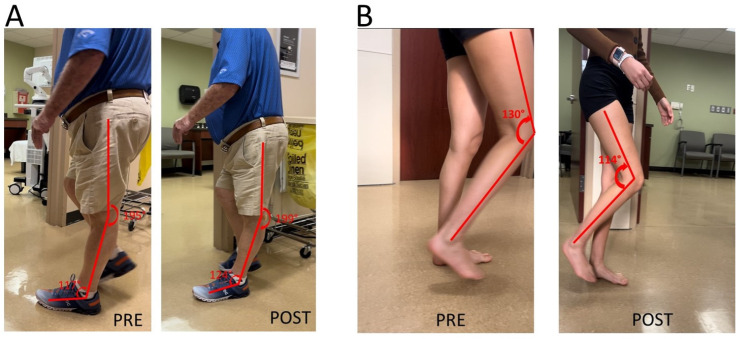
(**A**): Knee hyperextension due to a combination of ankle plantar flexor spasticity and knee extensor spasticity. worsening of knee hyperextension is observed after a lidocaine block to the tibial nerve. (**B**): Difficult knee flexion due to quadriceps muscle spasticity. Improvement in knee flexion during the swing phase is observed after a lidocaine block to femoral nerve motor branches to quadriceps muscles.

Knee extensor spasticity is commonly considered the primary cause of post-stroke stiff knee gait. Their clinical presentation of knee hyperextension during the stance could be similar to those caused by ankle plantar flexor spasticity in Figure 1. This was a case of a 71-year-old male with left hemiplegia and abnormal gait from right thalamic hemorrhage 5 years ago (Figure 2A). He was able to ambulate without any assistive device for 3 miles every day. He had near normal muscle strength except left hip flexors (3/5 on MMT). MAS was 1 for left knee extensors and 1+ for ankle plantar flexors. MAS was 0 for other muscles. Patellar tendon reflex was hyperreflexive on the left, but no ankle clonus was appreciated. He had a similar but lesser degree of gait abnormality as seen in the previous case (Figure 2A PRE). A diagnostic lidocaine block (1%) to the tibial nerve motor branches of the gastrocnemius and soleus muscles made knee hyperextension worse (Figure 2A POST). This outcome suggested that knee extensor spasticity plays an important role in the abnormal presentation of knee hyperextension in this case.

It is thus expected that addressing quadriceps muscle spasticity would improve stiff knee gait. Such improvement is demonstrated in the next case in Figure 2B. She was a 28-year-old woman with right spastic hemiplegia and abnormal gait from left frontal and parietal intracranial hemorrhage 3.5 years ago. She was able to ambulate without any device. She received periodic botulinum toxin injections to right arm muscles, but not leg muscles. However, she had difficulty flexing her knee during the swing phase, and her right knee snapped back during the stance phase. On exam, she had near normal motor strength except right ankle dorsiflexors flexors (1/5 on MMT). MAS was 1+ for right knee extensors, 1 for right ankle plantar flexors, and 1 for tibialis posterior muscle. Patellar tendon reflex was hyerreflexive on the right, normal on the left. After lidocaine diagnostic blocks (1%) to the femoral nerve motor branches of the rectus femoris, vastus medialis and vastus lateralis muscles, she was able to flex her right knee better during the swing phase, and no more “snapping back” during the stance phase.

### 2.3. Knee Extensor and Flexor Spasticity and Coactivation

**Figure 3:**
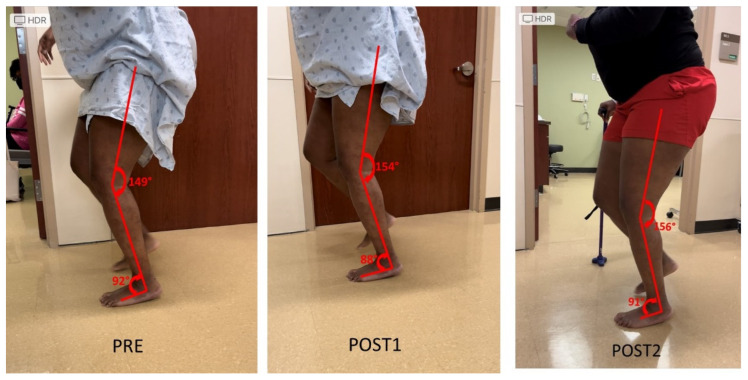
Persistent knee flexion throughout the gait cycle. Lidocaine blocks to the tibial nerve (POST1) or the tibial nerve and sciatic nerve motor branches to hamstrings muscles (POST2) do not have dramatic effects on knee motion during the gait cycle (PRE).

Knee extensor and flexor spasticity and coactivation can also limit the knee joint range of motion during gait. This case was a 36-year-old female with left spastic hemiplegia and abnormal gait from right middle cerebral artery (MCA) ischemic stroke 5.5 years ago (Figure 3). She ambulated with a point cane for safety. Her left leg was stiff during walking, acting like a stick except a few degrees of ankle motion during the stance phase. On exam, motor strength testing of left leg muscles was 3/5 for hip flexors, 3/5 for hip extensors, 4/5 for knee extensors, 3/5 for hamstrings, 4/5 for ankle plantar flexors, and 0/5 for ankle dorsiflexors. MAS was 1+ for hip flexors, 1+ for knee extensors and 1+ for hamstrings, 2 for ankle plantar flexors, 1 for tibialis posterior, 1+ for flexor digitorum longus muscle. Ankle clonus was ++ on the left. She had two diagnostic lidocaine blocks on different days. The first block was to the tibial nerve only (Figure 3 POST1), whereas the second block was to the tibial nerve and sciatic nerve branches to hamstrings a few days later (POST2). As seen in Figure 3, both blocks did not make dramatic changes to the knee and ankle joint during walking as compared to other cases.

### 2.4. Hip Flexor Spasticity

**Figure 4:**
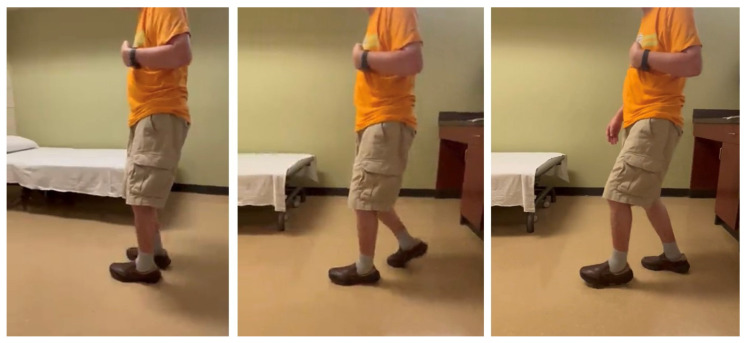
A step-to gait due to hip flexor spasticity in a patient with left spastic hemiplegia (walking from right to left).

Stiff knee gait can present as a mechanical consequence of hip flexor spasticity. In this case of a 69-year-old male with left spastic hemiplegia and abnormal gait from right MCA ischemic stroke 7 years ago, he had a “step-to” gait. As shown in Figure 4 (from right to left), his left leg advanced, while his right leg followed, and stepped to the left leg. On exam, muscle strength on the left leg was 3/5 for hip flexors, 2/5 for hip extensors, 4/5 for knee extensors, 3/5 for hamstrings, 2/5 for ankle plantar flexors, 0/5 for ankle dorsiflexors. MAS was 2 for hip flexors, 0 for knee extensors, 1 for hamstrings, 1+ for ankle plantar flexors. He received periodic botulinum toxin injections to his left arm muscles, but only to hip flexors recently (75 units of onabotulinum toxin A to iliopsoas muscles). He reported that his walking was much smoother and easier after injections to hip flexors. His hip flexor spasticity and weak hip extensor muscles prevented his left hip from a hip neutral or extension position during the left leg stance phase. He had some degrees of left hip flexion during the swing phase. However, due to persistent hip flexion throughout the gait cycle, his left knee maintained a similar degree of knee flexion to compensate for this mechanical misalignment.

To note, a different pattern of “step-to” gait is often observed. In a commonly observed circumductory gait, spastic hemiplegic patients usually have hip extensor spasticity and hip flexor weakness. To compensate for these motor impairments, patients usually rotate their trunk forward to advance the paretic leg. Similarly, the knee joint on the paretic side is often locked in the extended position to compensate for this mechanical misalignment and to maintain stability during the stance phase. Nevertheless, in both “step-to” gait patterns, knee joint motion is limited as a mechanical consequence of hip joint abnormality.

## 3. Discussion

The above cases clearly demonstrate various underlying mechanisms of SKG in stroke survivors with spastic hemiplegia. In addition to the well-accepted mechanism of knee extensor spasticity or co-contraction of knee flexors and extensors that directly limit knee flexion in the swing phase, other mechanisms, including hip flexor spasticity and ankle plantar flexor spasticity, indirectly affect knee joint movement during walking through kinetic chain reactions. These various clinical presentations of SKG in spastic hemiplegia reflect that SKG is a mechanical consequence between muscle weakness, spasticity and their interactions with the ground reaction forces [13].

Muscle spasticity and weakness are hallmark motor impairments in chronic stroke survivors [20]. Spasticity is present in up to 97% of stroke survivors with moderate to severe motor impairment [21]. Spasticity and weakness have different levels of severity and involve different areas of the limb, thus producing a full spectrum of motor impairment in stroke survivors and various presentations of stiff knee gait disorders. For example, there was ankle plantar flexor spasticity and hip flexor weakness in the first case (Figure 1). Ankle plantar flexor spasticity and its associated involuntary ankle plantar activation place the ankle into an abnormal joint position (ankle plantar flexion) during the midstance phase. This abnormal ankle joint position shifts the center of gravity anterior to the hip and knee joint, especially when the weak hip flexors is not able to move the femur forward. As a result, knee hyperextension occurs in the midstance, and not enough knee flexion torque is generated during the swing phase. On the other hand, knee hyperextension in the midstance phase can present as a direct result of overactivity of knee extensors (Figure 2A). As previously reported, co-activation of knee flexors and extensors can provide knee joint stability to compensate for knee extensor weakness during the midstance phase [19], although it limits knee flexion during the swing phase as well (Figure 3). Instrumented gait analysis with electromyographic (EMG) recordings from lower limb muscles are needed to confirm the neuromechanical analysis mentioned above. However, dramatic changes in joint alignment after lidocaine diagnostic tests are also confirmatory.

Understanding of these various mechanisms of SKG in chronic stroke is helpful to guide clinical assessment and to select the appropriate target muscles for interventions, especially because knee extensor spasticity is commonly accepted as the primary cause. Careful consideration of different causes and thorough clinical assessment are needed for appropriate diagnosis of the underlying mechanism. Lidocaine diagnostic tests are often recommended and used to differentiate and confirm the diagnosis in some cases, as described in Figure 1 and Figure 2A. Furthermore, the diagnostic tests can inform the target muscles for botulinum toxin injection or nerve block for the best clinical outcome (Figure 1).

As mentioned earlier, current clinical management of SKG focuses on spasticity reduction in the quadriceps muscles [7,9,10,11,12]. Understanding of these various mechanisms can shed light on alternative approaches for clinical management of SKG. In some cases, stiff-knee gait disorders could present as a compensatory gait pattern, such as in Figure 3, to provide knee joint stability during walking. As such, there is no need to disturb the well-compensated “abnormal” gait. If the compensated pattern is disturbed, the patient may need external assistance for stability as in Figure 3 (POST2). In other cases, the primary cause needs to be addressed, e.g., ankle plantar flexor spasticity (Figure 1) and hip flexor spasticity (Figure 4), so that the compensatory SKG is likely to improve.

## 4. Conclusions

Stiff knee gait in chronic stroke has various clinical presentations. They are mechanical consequences between muscle spasticity, weakness, and their interactions with ground reactions during walking. Careful and thorough clinical assessment is advised to determine the primary cause for each patient. Understanding of these various presentations is crucial to guide clinical assessment and select appropriate target muscles for interventions.

## Data Availability

The datasets used to support the findings of this study are available from the corresponding author upon request.
